# Cardiac imaging in Chagas cardiomyopathy for phenotypic characterisation and risk stratification

**DOI:** 10.1007/s10741-026-10645-z

**Published:** 2026-06-08

**Authors:** Anisha Johnson, Sai Vamshi Krishna Katraj, Vivetha Pooranachandran

**Affiliations:** 1 Department of Natural Sciences, Middlesex University, The Burroughs, London, NW4 4BT UK; 2 Medway NHS Foundation Trust, Kent, UK; 3 Sheffield Teaching Hospital, Sheffield, UK

**Keywords:** Chagas disease, *Trypanosoma Cruzi*, Cardiomyopathy, Cardiac MRI, Echocardiography, Strain imaging

## Abstract

Chagas disease, caused by *Trypanosoma cruzi*, remains one of the leading infectious causes of non-ischemic cardiomyopathy worldwide. Although an estimated 6–7 million individuals are chronically infected, 20–30% will develop Chagas cardiomyopathy (CCM), a progressive myocardial disease characterised by conduction abnormalities, ventricular arrhythmias, focal aneurysm formation, diffuse fibrosis, and heart failure. Cardiac involvement often evolves silently over decades, and conventional clinical markers frequently underestimate early myocardial injury. Advances in cardiac imaging have fundamentally reshaped the understanding of CCM, demonstrating that myocardial involvement frequently precedes symptoms and may occur despite normal electrocardiography. Contemporary modalities, including transthoracic echocardiography with deformation imaging and cardiac magnetic resonance, enable detailed characterisation of ventricular mechanics, myocardial fibrosis, inflammation, and perfusion abnormalities across disease stages. These techniques have revealed a distinctive, segmental pattern of myocardial injury and provided mechanistic insight into arrhythmogenesis and disease progression. This review synthesises current evidence on the natural history of Chagas disease, with a particular focus on the expanding role of multimodality cardiac imaging in the early detection, phenotypic characterisation, and risk stratification of CCM.

## Introduction

Chagas disease, first described in 1909 by Carlos Chagas, is caused by *Trypanosoma cruzi* (*T. cruzi)* and remains a leading cause of non-ischemic cardiomyopathy worldwide. An estimated 6–7 million individuals are currently infected, and approximately 20–30% will develop Chagas cardiomyopathy (CCM) over time. While historically confined to Latin America, particularly the Southern Cone, Central America, and parts of Mexico, migration has led to increasing recognition of Chagas disease in non-endemic regions, including the United States, Canada, Spain, and Italy, where it poses diagnostic challenges in cardiology, transplant, and obstetric settings [1].

Triatomines are nocturnal hematophagous (blood-sucking) insects of the Reduviidae family, commonly known as “kissing bugs.” Their original habitat consists of cracks in walls, thatched roofs, and peridomestic structures in rural and semi-rural areas of Latin America, where they colonise human dwellings and animal shelters, facilitating vector-borne transmission of T. *cruzi* [1]. Transmission occurs predominantly via triatomine vectors in endemic regions, while congenital transmission, blood transfusion, and organ transplantation account for most cases in non-endemic countries [2]. Congenital transmission affects approximately 1–10% of infants born to infected mothers, contributing to multigenerational cardiac risk. Because cardiomyopathy typically develops decades after initial infection, patients often present in middle age with limited awareness of early exposure [3].

The acute phase of Chagas disease is frequently oligosymptomatic but may include clinically significant cardiac involvement, particularly in outbreaks related to oral transmission, which are associated with higher parasitic burden and more intense myocardial inflammation. Documented manifestations include transient myocarditis, pericardial effusion, and arrhythmias [4]. In a prospective series of 52 patients with acute Chagas myocarditis, pericardial effusion occurred in 42%, usually mild to moderate but occasionally severe [5]. Although acute systolic dysfunction is uncommon and most patients recover clinically, acute cardiac involvement is prognostically relevant: individuals with documented acute myocarditis demonstrate accelerated progression to chronic CCM, with an estimated annual risk of approximately 4.6%, compared with ~ 1.9% among those without acute cardiac involvement [6].

Following the acute phase, infection enters a chronic stage that begins 4–8 weeks after exposure and persists indefinitely. This stage encompasses three clinical forms: indeterminate, cardiac, and digestive. The indeterminate form, defined by normal ECG, chest radiography, and digestive studies, accounts for approximately 60% of chronically infected individuals and may persist for decades [7]. Importantly, CCM refers to the full spectrum of myocardial involvement caused by *T. cruzi*, including electrical abnormalities, arrhythmias, structural remodelling, and ventricular dysfunction, which may occur with or without symptoms. Heart failure represents a late clinical manifestation of CCM rather than a synonym for cardiomyopathy itself [8]. Longitudinal cohorts demonstrate that 30–40% of chronically infected individuals ultimately develop cardiac manifestations, reflecting ongoing myocardial injury driven by parasite persistence, microvascular dysfunction, autonomic imbalance, and chronic inflammation [7].

Progression from the indeterminate form to established CCM is typically gradual but unpredictable. Ventricular arrhythmias and conduction abnormalities often represent the earliest manifestations of cardiac disease and may remain clinically silent for prolonged periods or present with palpitations, syncope, or sudden cardiac arrest [9]. Sudden cardiac death (SCD) is a major cause of mortality in CCM and may occur at any disease stage, sometimes preceding the development of symptomatic heart failure. As disease advances, a subset of patients progress to overt ventricular dysfunction and heart failure. The A–D staging system captures this continuum, ranging from asymptomatic infection without detectable cardiac disease (Stage A), through subclinical and early structural involvement (Stages B1 and B2), to symptomatic heart failure (Stages C and D). Once CCM progresses to symptomatic heart failure, outcomes worsen substantially, with pooled analyses indicating annual mortality rates of ~ 7.9%, rising to ~ 22% per year in advanced stages [6].

Early identification of *T. cruzi* infection is essential for preventing progression and interrupting transmission. Diagnosis of chronic Chagas disease relies on serologic confirmation using at least two distinct assays, as no single test provides sufficient accuracy. ELISA, indirect haemagglutination, and immunofluorescence tests are most commonly used in combination [10–12]. In contrast, acute Chagas disease is diagnosed through direct parasitological methods, such as microscopic examination of peripheral blood or concentration techniques, because parasitaemia is high during early infection. Polymerase chain reaction (PCR) is also highly sensitive in acute disease and is increasingly used to confirm infection and monitor treatment response. Recognition of acute infection is clinically important, as acute myocarditis may occur and is associated with faster progression to chronic cardiomyopathy [10–12].

Once infection is confirmed, systematic cardiac evaluation is mandatory, as electrical abnormalities often represent the earliest clinically detectable manifestation of CCM. A 12-lead ECG is universally recommended as the first-line cardiac test [13]. A large meta-analysis of 49 population-based studies including more than 34,000 participants demonstrated that ECG abnormalities are present in 40.1% of individuals with Chagas disease, compared with 24.1% of non-infected controls. Characteristic findings include right bundle branch block, left anterior fascicular block, atrioventricular block, and ventricular ectopy. These abnormalities are not merely diagnostic but carry prognostic significance: progression from a normal ECG to conduction disease marks the transition from indeterminate infection to cardiac involvement and is associated with a two- to three-fold increase in all-cause mortality. Ventricular arrhythmias further confer increased risk of SCD, even in the absence of overt heart failure [14].

However, while ECG serves as an essential gatekeeper, it cannot characterise myocardial structure, fibrosis, inflammation, or regional mechanics [6]. Consequently, cardiac imaging is required to define disease phenotype, stage myocardial involvement, and refine risk assessment. The sections that follow review the complementary roles of cardiac imaging, and emerging artificial intelligence approaches within a stage-based framework for CCM (Table 1).

**Table 1:** Comparative strengths, limitations, and clinical roles of cardiac imaging modalities in Chagas cardiomyopathy

Imaging Modality	Primary Purpose	Strengths	Limitations	Best Clinical Use
Chest X-ray	Structural overview; prognosis	Widely available; low cost; incorporated into Rassi score; detects cardiomegaly and thoracic complications	Low sensitivity for early disease; no functional or tissue characterisation	Initial evaluation; prognostication in advanced disease
Echocardiography	Functional phenotyping	Detects regional dysfunction, aneurysms, thrombus; strain and myocardial work detect early dysfunction; bedside availability	Limited tissue characterisation; operator dependent; acoustic window limitations	First-line cardiac assessment; longitudinal follow-up
Cardiac Magnetic Resonance	Myocardial characterisation	Gold standard for ventricular volumes; detects focal and diffuse fibrosis (late gadolinium enhancement, T1, extracellular volume); identifies arrhythmogenic substrate; detects early disease	Limited availability; contraindications; cost	Disease staging; risk stratification; arrhythmia substrate assessment
Cardiac CT	Anatomic clarification	Excludes obstructive coronary artery disease; detects aneurysms and focal fibrosis; quantifies ventricular volumes	Radiation exposure; limited tissue characterisation	Alternative when CMR contraindicated; coronary assessment
PET Scan	Inflammatory activity	Detects active myocardial inflammation; identifies scar–inflammation interface; guides VT ablation	Limited availability; lack of outcome cohorts; technical complexity	Selected cases with ventricular arrhythmias or unexplained progression
AI-based tools (ECG/Echo)	Screening and risk enrichment	Scalable; low cost; detects left ventricular dysfunction and mortality risk	Indirect myocardial assessment; limited imaging integration	Population screening; triage for advanced imaging

*CMR *Cardiac Magnetic Resonance,* CT* Computer Tomography, *PET* Positron Emission Tomography, *AI* Artificial Intelligence, *ECG* Electrocardiogram, *Echo* Echocardiogram, *VT* Ventricular Tachycardia

## Imaging in Chagas cardiomyopathy

### Chest radiography

Chest X-ray remains a readily available and low-cost tool in the initial evaluation of patients with chronic *T. cruzi* infection and continues to play a complementary role in assessing cardiac involvement, particularly in resource-limited settings [15]. However, its utility in early disease detection is limited. Many patients with significant systolic dysfunction, regional wall-motion abnormalities, or myocardial injury detectable by echocardiography or cardiac magnetic resonance (CMR) may exhibit only mild or absent radiographic changes, especially during the indeterminate phase [16]. Observational cohorts and systematic reviews consistently demonstrate that chest radiographic abnormalities often lag behind functional impairment, underscoring its low sensitivity for early cardiomyopathic involvement [17–19].

Phenotypically, chest radiography provides only global structural information, most notably through assessment of the cardiothoracic ratio (CTR). In advanced CCM, posteroanterior radiographs typically demonstrate cardiomegaly reflecting progressive chamber enlargement [18]. In a retrospective study of 63 Chagas outpatients, 44% (28/63) exhibited cardiomegaly on chest X-ray. Using echocardiography as the reference standard, a left ventricular end-diastolic diameter ≥ 60 mm best corresponded to the presence of cardiomegaly on chest X-ray, with approximate moderate sensitivity of 64% but high specificity of 89% (area under the curve (AUC) 0.806), indicating that chest radiography reliably identifies advanced ventricular enlargement but frequently fails to detect earlier stages of remodelling [18].

Despite its limited role in early detection and phenotypic characterisation, chest X-ray has robust prognostic value in CCM. An elevated CTR (> 0.50) has been validated as an independent predictor of mortality, correlating with advanced ventricular remodelling and worse long-term outcomes [19]. This prognostic relevance is most clearly demonstrated by its inclusion in the Rassi score, the most widely validated risk stratification model for chronic CCM. Derived from a cohort of 424 Brazilian outpatients followed for a mean of 7.9 years, the Rassi score assigns points to six independent predictors of mortality, including cardiomegaly on chest X-ray (5 points), placing it on par with advanced NYHA functional class and above several electrocardiographic variables. Stratification into low, intermediate, and high-risk categories predicted 10-year mortality rates of approximately 10%, 44%, and 84%, respectively, with strong discrimination in both derivation and validation cohorts [20].

The prognostic weight of cardiomegaly on chest radiography has also influenced patient selection in device trials. Notably, the CHAGASICS trial relied on the Rassi score, incorporating chest X-ray cardiomegaly and echocardiographic systolic dysfunction, rather than advanced imaging phenotypes. While implantable cardioverter-defibrillator (ICD) therapy reduced SCD, it failed to improve all-cause mortality, likely reflecting residual risk from progressive myocardial disease not captured by conventional markers [21]. These findings highlight the limitations of chest radiography–based risk stratification and underscore the need for advanced imaging-guided approaches.

### Echocardiography

Echocardiography plays a central role in the evaluation of CCM by enabling early detection of cardiac involvement, often before the onset of symptoms or global systolic dysfunction. In contrast to other non-ischemic cardiomyopathies, Chagas disease is characterised by early, regional, and highly localised ventricular abnormalities, which frequently precede any measurable decline in left ventricular ejection fraction (LVEF) [7]. Segmental wall-motion abnormalities, most commonly involving the inferolateral, apical, and basal segments, reflect the patchy distribution of parasite-driven inflammation and fibrosis and may be evident even when conventional measures of systolic function remain normal [22]. Importantly, echocardiographic abnormalities in Chagas disease are not diagnostic in isolation and must be interpreted in conjunction with epidemiologic exposure and serologic confirmation of *T. cruzi* infection. Rather than serving as a substitute for serology, echocardiography functions as the principal tool for identifying and confirming cardiac involvement once infection is established [23].

Beyond detection, echocardiography provides critical insights into the distinctive myocardial phenotype of CCM. A left ventricular apical aneurysm, reliably detected by echocardiography, is a near-pathognomonic feature in the appropriate epidemiologic context and is rarely encountered in other non-ischemic cardiomyopathies [24]. CCM is also marked by a disproportionately high burden of intracardiac thrombus [25]. In a clinicopathologic review of 12 studies involving 431 patients, right-sided intracardiac thrombus was identified in 66–85% of autopsied cases with pulmonary embolism. Notably, even asymptomatic individuals with isolated right bundle branch block demonstrated right ventricular dilation, right ventricular systolic dysfunction, right atrial enlargement, and large intracavitary thrombi detectable only through echocardiography [26].

Structural remodelling in Chagas disease follows a trajectory distinct from other forms of dilated cardiomyopathy, with changes in left ventricular dimensions emerging early in the disease course [27]. This pattern was recapitulated in a natural infection canine model of 130 PCR-positive dogs, of whom 11.5% developed overt dilated cardiomyopathy. Increases in left ventricular internal diameters, posterior wall thinning, interventricular septal abnormalities, and reductions in fractional shortening were the strongest discriminators of progression. Importantly, even infected animals without clinical cardiomyopathy exhibited early interventricular septal and posterior wall abnormalities, supporting the concept that subclinical geometric remodelling is a hallmark of early CCM [28].

Human longitudinal studies reinforce the prognostic value of both conventional and advanced echocardiographic indices in CCM. In a landmark cohort of 856 patients, left ventricular end-systolic diameter independently predicted long-term mortality, underscoring the importance of tracking progressive remodelling [29]. Among patients with early CCM, defined by abnormal ECG findings or segmental wall-motion abnormalities in the absence of heart failure, Cunha et al. followed 176 patients for a mean of 8.8 years to evaluate progression to symptomatic heart failure. Incident heart failure was predicted not only by modest reductions in LVEF but also by markers of early myocardial dysfunction, including lower mitral annular E′ velocity, impaired circumferential strain, and abnormal left atrial booster strain. Importantly, deformation-based indices remained independently associated with heart failure development after adjustment for conventional echocardiographic parameters, demonstrating incremental prognostic value in identifying patients at risk of progression despite preserved or near-preserved systolic function [30].

In more advanced disease, Maia et al. evaluated 317 patients and showed that although mean LVEF was modestly reduced (41 ± 12%), global longitudinal strain (GLS) was a substantially stronger predictor of mortality than LVEF, left atrial volume, E/e′ ratio, or right ventricular size. GLS was particularly informative in patients with preserved or mildly reduced LVEF, consistent with the segmental nature of Chagas myocardial injury [23]. Emerging echocardiographic techniques further extend early disease detection: in a cohort of 50 patients across stages A–C, myocardial work indices (Global constructive work, Global work index, and Global work efficiency) were significantly reduced in patients with early systolic dysfunction, with global constructive work demonstrating excellent discriminative accuracy for identifying early left ventricular systolic impairment (AUC 0.976; *r* = 0.854 with LVEF) [31].

Taken together, echocardiography in Chagas disease does not merely document generic cardiomyopathy but delineates a distinctive, segmental, and progressive myocardial phenotype that reflects parasite-driven inflammation, microvascular injury, and autonomic imbalance. While echocardiographic findings alone do not dictate antiparasitic or device-based therapy, they play a pivotal role in detecting cardiac involvement, defining disease phenotype, and stratifying risk for heart-failure progression, arrhythmias, and mortality, thereby guiding surveillance strategies, adjunctive testing, and individualised clinical management.

### Cardiac Magnetic Resonance 

CMR is regarded as the gold-standard non-invasive imaging modality for detecting myocardial involvement in Chagas disease, owing to its ability to integrate high-resolution assessment of ventricular morphology, systolic function, regional mechanics, and myocardial tissue composition [32]. Critically, CMR identifies myocardial abnormalities in up to 20% of seropositive, asymptomatic individuals with normal ECG and echocardiography [33]. Across the disease spectrum, CMR can detect regional wall-motion abnormalities, subtle ventricular systolic dysfunction, apical aneurysm formation, myocardial oedema, focal replacement fibrosis, and diffuse interstitial remodelling, abnormalities that often precede changes detectable by conventional imaging [32].

The incremental sensitivity of CMR over ECG and echocardiography was demonstrated in a retrospective cohort of 81 untreated Chagas patients from a Latin American migrant population in the United States. Among patients with normal ECG and echocardiography, 8% already exhibited delayed myocardial enhancement, while the prevalence increased to 30% in those with abnormal ECG but normal echocardiography and 67% in those with echocardiographic abnormalities [34]. These findings confirm that CMR frequently detects myocardial injury at a stage when conventional testing remains normal, redefining the earliest detectable phase of cardiac involvement.

Beyond detection, CMR provides unparalleled phenotypic characterisation of CCM. A hallmark feature is myocardial fibrosis, most commonly identified using late gadolinium enhancement (LGE). The role of LGE in disease staging was established in a seminal prospective study by Rochitte et al., who evaluated 51 patients across the disease spectrum (indeterminate disease, established cardiomyopathy, and cardiomyopathy with ventricular tachycardia). Myocardial fibrosis was detected in 68.6% overall, with a striking stepwise increase according to disease severity (20%, 84.6%, and 100%, respectively; *p* < 0.001). Fibrosis burden increased progressively and correlated strongly with both global and regional systolic dysfunction, establishing LGE-CMR as a quantitative marker of myocardial injury and arrhythmogenic substrate, detectable even in asymptomatic stages [35].

Subsequent studies confirmed that LGE in CCM exhibits a distinctive, non-ischemic and heterogeneous distribution, with predilection for the apical and inferolateral segments [32]. In a European cohort of 67 Chagas patients living in a non-endemic region, delayed enhancement demonstrated mixed subendocardial, mid-wall, subepicardial, and transmural patterns, correlating with larger ventricular volumes and worse systolic function [36]. This heterogeneous enhancement pattern, sometimes mimicking ischemic and non-ischemic cardiomyopathies, highlights the diagnostic value of CMR in patients with compatible epidemiologic exposure and heart failure of uncertain aetiology [37].

### Diffuse fibrosis, arrhythmias, and clinical outcomes

While LGE identifies focal replacement fibrosis, it underestimates diffuse interstitial fibrosis, a central pathological feature of chronic *T. cruzi* infection [32]. Parametric mapping techniques, including native T1 and extracellular volume (ECV) mapping, have addressed this limitation [38]. In a prospective longitudinal study of 107 participants (90 Chagas patients and 17 controls), global and remote (LGE-negative) T1 and ECV values increased progressively with disease severity. Markedly elevated ECV values (~ 35%) were observed in patients with mid-range and reduced ejection fraction, compared with ~ 25% in indeterminate disease and controls (*p* < 0.001). Notably, 12% of indeterminate patients already demonstrated abnormal remote ECV (> 30%), indicating diffuse myocardial involvement preceding both LGE and systolic dysfunction [39].

Importantly, these diffuse fibrosis markers carry prognostic significance. During a median follow-up of 43 months, a remote native T1 value > 1100 ms independently predicted major adverse cardiovascular events, including ICD implantation, heart transplantation, or death, with a hazard ratio of 12.0 (95% CI 4.1–34.2; *p* < 0.001) [39]. Arrhythmic risk associated with diffuse fibrosis has also been demonstrated in early-stage disease. In a cross-sectional cohort of 47 patients with early Chagas disease, ECV remained independently associated with non-sustained ventricular tachycardia, even after adjustment for fibrosis mass and LVEF, and showed strong discriminatory performance for arrhythmic risk (AUC 0.85), numerically outperforming LGE-derived fibrosis burden [40].

The sensitivity of CMR for detecting subclinical myocardial injury has been corroborated in naturally infected canine models that closely mirror human disease. In a prospective observational study of 10 asymptomatic, client-owned dogs seropositive for *T. cruzi*, abnormalities were detected most frequently by CMR (7/10 dogs), exceeding ambulatory ECG, echocardiography, standard ECG, and cardiac biomarkers. Findings included delayed myocardial enhancement, increased ECV, regional wall-motion abnormalities, and loss of apical compact myocardium, reinforcing the role of CMR as the most sensitive modality for early myocardial involvement and validating these models for translational research [33].

Collectively, these studies demonstrate that CMR provides unique, pathophysiologically grounded information across the full spectrum of Chagas disease, from early diffuse fibrosis and subclinical myocardial involvement to advanced scar burden, ventricular dysfunction, and arrhythmogenic substrate [32]. Despite strong associations with arrhythmias, heart-failure progression, and hard clinical outcomes, CMR-derived markers remain absent from validated risk scores and ICD selection algorithms, which continue to rely on clinical variables, chest X-ray cardiomegaly, and echocardiographic LVEF. This disconnect likely contributes to the observed reduction in SCD without improvement in all-cause mortality in device trials [21].

### Computed tomography and positron emission tomography

Computed tomography (CT) and positron emission tomography (PET) serve as adjunctive imaging modalities in the evaluation of CCM, offering complementary anatomic and functional insights that extend beyond conventional echocardiography and CMR [41]. While large prospective outcome cohorts are limited, evidence from multimodality imaging studies, case-based reports, and expert consensus documents demonstrates that CT and PET contribute meaningfully to etiologic clarification, detection of myocardial fibrosis and inflammation, and assessment of arrhythmogenic substrate, particularly in complex or advanced disease [42].

### Cardiac CT: coronary exclusion, fibrosis detection, and ventricular morphology

Cardiac CT is performed using iodine-based contrast with ECG synchronisation, employing either prospective acquisition (lower radiation dose, diastolic imaging) or retrospective acquisition (higher radiation dose with full functional assessment) [43]. According to the ASE/ECOSIAC expert consensus on multimodality imaging of neglected tropical diseases, cardiac CT is particularly useful in Chagas disease for excluding obstructive coronary artery disease, defining ventricular morphology, and identifying apical aneurysms, especially when echocardiography is limited or CMR is contraindicated [44].

The clinical value of CT angiography in CCM is illustrated in a multimodality imaging case report of a 58-year-old man with chronic CCM and severe left ventricular dysfunction. In this report, coronary CT angiography demonstrated normal epicardial coronary arteries, effectively excluding ischemic cardiomyopathy in a patient with advanced heart failure and ischemic-appearing features. Importantly, delayed iodine enhancement CT revealed transmural enhancement in inferolateral and apical aneurysmatic segments, corresponding to areas of myocardial fibrosis later confirmed by CMR. CT also demonstrated four-chamber dilation and severe biventricular systolic dysfunction, complementing echocardiographic findings and contributing to disease staging [45].

Although systematic sensitivity estimates are lacking, this and similar reports support the concept that contrast-enhanced CT can identify focal myocardial fibrosis and aneurysmal remodelling in CCM, providing a viable alternative when CMR is unavailable [32]. CT additionally allows quantitative assessment of left and right ventricular volumes and systolic function, which may enhance diagnostic confidence when echocardiographic windows are suboptimal [46].

### PET imaging: myocardial inflammation and arrhythmogenic substrate

While CT provides detailed anatomical information, PET imaging uniquely enables in vivo assessment of myocardial inflammation, a key pathophysiological process in chronic *T. cruzi* infection [32]. Persistent low-grade myocarditis and immune activation have been implicated in disease progression and arrhythmogenesis, particularly at the interface between viable myocardium and fibrotic scar [9].

Direct evidence of PET utility in CCM is currently limited to case-based and mechanistic studies, but these provide important proof of concept. In a hybrid PET/CT case study of a 68-year-old man with Chagas disease and sustained ventricular tachycardia, ¹⁸F-FDG PET/CT and ⁶⁸Ga-DOTATOC PET/CT demonstrated focal tracer uptake adjacent to hypoperfused and fibrotic myocardial regions previously identified by CMR. These findings indicated active myocardial inflammation bordering established scar, supporting the hypothesis that ongoing inflammatory activity contributes to ventricular tachycardia initiation and maintenance [47].

Notably, ⁶⁸Ga-DOTATOC, which targets somatostatin receptor–expressing inflammatory cells and does not exhibit physiological myocardial uptake, provided improved specificity compared with FDG. This tracer has been extensively studied in cardiac sarcoidosis and, as shown in this Chagas case, may represent a promising tool for identifying arrhythmia-prone inflammatory substrate in inflammatory cardiomyopathies [42].

### Integration with arrhythmic risk stratification and interventions

The integration of CT and PET findings with echocardiography and CMR offers a multimodality framework that captures coronary anatomy, focal fibrosis, ventricular remodelling, and active inflammation [48]. In the reported PET/CT case, the identification of inflammatory activity adjacent to fibrotic scar provided mechanistic insight into sustained ventricular tachycardia, supporting aggressive management including ICD implantation and informing considerations for ventricular tachycardia ablation planning [47].

The ASE/ECOSIAC consensus document acknowledges the emerging role of nuclear imaging techniques, including PET, for identifying myocardial inflammation and perfusion abnormalities in Chagas disease, particularly in patients with ventricular arrhythmias or discordant findings on echocardiography and CMR. However, it also emphasises that CT- and PET-derived markers are not yet incorporated into validated prognostic scores or guideline-based ICD selection algorithms, reflecting the absence of large, longitudinal outcome studies [44].

Despite their demonstrated ability to identify critical pathophysiological processes, including coronary anatomy, aneurysm formation, focal fibrosis, and active inflammation, CT and PET remain adjunctive tools in CCM. Their prognostic value has not been quantified in prospective cohorts, and standardised thresholds for risk stratification are lacking [32]. Nevertheless, by addressing domains not fully captured by echocardiography or CMR, particularly active inflammation and scar–inflammation interaction, CT and PET represent important candidates for future imaging-guided risk models and device-selection strategies.

### Artificial intelligence and advanced computational approaches

Artificial intelligence (AI) based methods are increasingly being explored as scalable tools for screening, risk stratification, and disease monitoring in Chagas disease, particularly in settings where access to advanced cardiac imaging remains limited [49]. While direct AI applications to cardiac imaging in Chagas disease are still sparse, growing evidence demonstrates that AI models applied to electrocardiography, echocardiography, and multimodal cardiac data can capture latent signatures of myocardial dysfunction that parallel imaging-defined structural and functional abnormalities [50]. These approaches position AI as both a surrogate marker of cardiac involvement and a triage mechanism guiding targeted use of imaging modalities.

### AI applied to ECG: indirect linkage to imaging-defined myocardial dysfunction

The strongest human evidence to date involves AI-enhanced ECG (AI-ECG) models for detecting left ventricular systolic dysfunction (LVSD), the most powerful predictor of mortality in CCM and a parameter traditionally defined by echocardiography or CMR [51, 52]. In a large prospective analysis derived from the SaMi-Trop cohort, which enrolled seropositive individuals from 21 municipalities in Minas Gerais, Brazil, AI-ECG models were fine-tuned and validated across multiple longitudinal assessments (baseline 2013–2014, follow-up 2015–2016 and 2022–2023). The model was trained on ECGs parsed into 2-second windows and achieved excellent discrimination for LVSD, with an AUC of 0.88 (95% CI 0.78–0.97) [51].

Crucially, AI-detected LVSD (AI-LVSD) demonstrated a stronger association with mortality than NT-proBNP [51]. AI-LVSD was associated with hazard ratios of 4.5 (95% CI 3.0–6.8) for two-year mortality and 4.3 (95% CI 3.4–5.5) for overall mortality. In simplified models incorporating only age, NYHA functional class, and AI-LVSD, the AI marker remained a dominant predictor of death (HR 5.4 for two-year and 4.9 for overall mortality) [51]. Although AI-ECG does not provide direct anatomical or tissue characterisation, these results indicate that AI-derived electrical features reflect imaging-defined ventricular dysfunction and disease severity, supporting its role as a digital surrogate for echocardiographic and CMR findings.

External validation in a resource-limited, real-world setting further reinforces this concept. In a retrospective study of 326 patients with Chagas disease in Venezuela, smartphone-captured paper ECGs were digitised and analysed using a deep-learning AI-ECG model. Among 293 analysable ECGs, the prevalence of reduced LVEF was 14.3% (< 50%) and 9.9% (≤ 40%), as defined by transthoracic echocardiography. The AI-ECG model achieved sensitivities of 90–94% and negative predictive values of 98–100% for detecting reduced LVEF, highlighting its effectiveness as a screening and rule-out tool for imaging-defined LV dysfunction [53].

### AI applied to echocardiography and multimodal data: direct linkage to imaging

Beyond ECG-based approaches, direct AI application to cardiac imaging has been explored in preclinical models. A recent experimental study using 96 ICR mice with *T. cruzi* infection investigated a hybrid AI framework combining machine learning (ML) and deep learning (DL) to classify Chagas disease using cardiac biomarkers and echocardiography video. From 64 cardiac biomarkers, an ensemble feature selection approach identified 17 highly informative features, which were used to train ML models. Weighted multiple kernel learning achieved 80% accuracy, 84% AUC, and 80% F1 score. Separately, DL models applied directly to echocardiography video data achieved more modest performance (65% accuracy, 60% AUC), reflecting the complexity of imaging-only classification in early disease [54].

Importantly, late multimodal fusion of ML-derived biomarker features and DL-based echocardiography outputs significantly improved diagnostic performance, achieving 84% AUC with 80% accuracy and F1 score [54]. This study provides direct proof-of-concept that AI can integrate echocardiographic imaging with biological data to enhance early detection of Chagas disease, demonstrating a pathway for future translation into human imaging cohorts [55].

Taken together, current evidence suggests that AI in Chagas disease operates along two complementary pathways. Indirectly, AI-ECG models detect electrical signatures that strongly correlate with imaging-defined LV dysfunction and prognosis, enabling population-level screening and prioritisation for imaging in resource-limited settings [56]. Directly, emerging AI approaches applied to echocardiographic imaging and multimodal cardiac data demonstrate the feasibility of automated image-based disease classification, albeit currently limited to experimental models [57].

Although AI-derived markers are not yet incorporated into established risk scores or imaging-guided decision algorithms, their strong association with imaging phenotypes and outcomes highlights their potential role as front-line digital biomarkers. Future integration of AI with echocardiography, CMR-derived fibrosis and strain metrics, and PET-based inflammatory imaging may enable precision risk stratification, bridging the gap between scalable screening tools and advanced multimodality imaging in CCM.

## Conclusion

Chagas cardiomyopathy remains a complex, progressive, and globally relevant non-ischemic cardiomyopathy. Modern imaging has revolutionised its detection and understanding, revealing myocardial involvement long before conventional markers such as ECG or LVEF decline. Echocardiographic strain imaging, CMR fibrosis characterisation, and CT/PET provide unprecedented insight into the structural, mechanical, and inflammatory substrates of disease. When integrated with biomarkers and genetic predictors, these modalities enable risk stratification and early identification of individuals at risk for severe complications.

As migration increases the prevalence of CCM in non-endemic regions, heightened awareness and systematic screening in cardiology practice are essential. Continued integration of advanced imaging, molecular diagnostics, and genetic insights will be critical in improving early detection and reducing the burden of this lifelong, often silent, and potentially fatal cardiomyopathy.

## Data Availability

No datasets were generated or analysed during the current study.
